# Exploring the Effects of a Computerized Naming Intervention Combined with Cerebellar tDCS in Cantonese-Speaking Individuals with Aphasia

**DOI:** 10.3390/brainsci16020137

**Published:** 2026-01-28

**Authors:** Maria Teresa Carthery-Goulart, Ada Chu, Anthony Pak-Hin Kong, Mehdi Bakhtiar

**Affiliations:** 1 Aphasia Research and Therapy (ART) Laboratory, Unit of Human Communication, Learning and Development, Faculty of Education, The University of Hong Kong, Hong Kong, China; carthery@hku.hk (M.T.C.-G.); ac616@hku.hk (A.C.); akong@hku.hk (A.P.-H.K.); 2 Speech and Neuromodulation Laboratory, Unit of Human Communication, Learning, and Development, Faculty of Education, The University of Hong Kong, Hong Kong, China

**Keywords:** aphasia, non-invasive brain stimulation, transcranial direct-current stimulation, cerebellum, stroke rehabilitation, computerized intervention, speech perception training, naming intervention, noun naming, verb naming

## Abstract

**Background/Objectives**: This study examined the effects of a computerized naming intervention combined with either cerebellar anodal transcranial direct-current stimulation (A-tDCS) or sham (S-tDCS) on noun and verb naming in Cantonese-speaking persons with chronic stroke-related aphasia (PWA). **Methods**: A double-blind, randomized, crossover, sham-controlled clinical trial was conducted with six Cantonese-speaking PWA following stroke. Participants received a 60 min computerized naming intervention incorporating audio–visual speech perception cues over five consecutive days, paired with concurrent 20 min of either 2 mA cerebellar A-tDCS or S-tDCS. Generalized linear mixed-effects models (GLMM) and linear mixed-effects models (LME) were used to evaluate naming accuracy and reaction time (RT). Individual variability was further explored through single-case analyses of naming accuracy changes across conditions and grammatical categories. **Results**: The GLMM showed a significant three-way interaction of condition, grammatical category, and time (*p* < 0.05). Specifically, the intervention paired with S-tDCS significantly improved verb naming, whereas A-tDCS did not induce significant improvements at the group level, effectively showing significantly smaller gains regarding verb naming compared to S-tDCS. Overall, RT decreased post-treatment across groups, but no significant differences emerged by the tDCS condition. The results support the promising efficacy of the Cantonese computerized audio–visual noun and verb naming therapy. Single-case analyses revealed high inter-individual variability in response to neuromodulation effects on naming and behavioral treatment outcomes. **Conclusions**: This study contributes to the emerging literature on cerebellar neuromodulation in post-stroke aphasia and underscores the need for larger trials examining grammar-specific (particularly verb-related) effects and polarity-dependent outcomes. It also highlights the value of developing personalized neuromodulation protocols to optimize the efficacy of behavioral language interventions in people with aphasia.

## 1. Introduction

Aphasia is a common sequela following stroke, affecting approximately one-third of survivors and resulting in profound emotional distress, activity limitations, and reduced social participation, all of which impair quality of life [1,2]. Effective interventions to address these language impairments are thus essential for improving functional outcomes and patient well-being. While speech–language therapy is well-established for improving language deficits, its efficacy is highly variable [3,4], and recovery often remains limited even with treatment [5,6]. Non-invasive brain stimulation (NIBS) techniques, such as transcranial direct-current stimulation (tDCS), have gained attention as adjuncts to behavioral therapies, with potential to improve post-stroke speech and language recovery by facilitating neural reorganization [7]. As an adjunctive intervention for aphasia, tDCS is valued due to its portability, low cost, ease of administration, and compatibility with speech–language pathology (SLP) practices, facilitating integration into routine clinical sessions to enhance therapy outcomes [8,9,10].

tDCS delivers low-intensity (1–2 mA) direct electrical currents to the scalp via electrodes, modulating neuronal firing rates through subthreshold changes in membrane potentials—depolarization (reducing the threshold for action potential firing) or hyperpolarization (increasing it)—thereby inducing lasting effects on synaptic plasticity and cortical excitability [11,12,13]. These effects are polarity-dependent: anodal tDCS (A-tDCS) enhances cortical excitability, whereas cathodal tDCS (C-tDCS) reduces it, exerting an inhibitory effect at the stimulated site [11].

Traditionally, cortical targets in the left-hemisphere language network have been favored for aphasia rehabilitation [9,10,14]. However, cortical stimulation presents limitations in brain-injured individuals, including altered conductivity due to post-stroke cystic encephalomalacia, where cerebrospinal fluid (CSF) accumulation distorts current flow to cortical targets [15,16,17]. These challenges have prompted investigation of alternative sites, particularly the cerebellum [18,19], historically associated with motor coordination and articulation but increasingly implicated in linguistic and cognitive functions [20,21] and often spared in PWA. Functional connectivity studies link the cerebellum to motor, linguistic, and executive networks (see [22] for a review). Neuroimaging evidence further reveals robust cerebellar involvement in language tasks, with right-posterolateral activations (lobules VI and VII, including Crus I/II) during verb generation, semantic processing, phonological encoding, and verbal working memory [23,24].

Despite its potential as stimulation site for post-stroke PWA, only a few studies have investigated cerebellar transcranial direct-current stimulation (tDCS) combined with language therapy for aphasia rehabilitation. Single-case designs and small-scale randomized controlled trials (RCTs) have addressed the effects of anodal or cathodal stimulation contrasted to sham controls, reporting inconsistent results regarding superior effects compared to sham and optimal stimulation polarity. For instance, Sebastian et al. [25] demonstrated in a single-case study that anodal cerebellar tDCS paired with spelling treatment significantly enhanced accuracy for both trained and untrained words in a participant with chronic post-stroke aphasia, with superior maintenance at 2 months and generalization to dictation and written picture naming compared to sham. In contrast, DeMarco et al. [26] found minimal language benefits in an exploratory study in which 10 PWA receiving anodal tDCS with multimodal naming therapy were compared to a 14 PWA sham control group, noting only an effect on mean utterance length in a picture description task. Marangolo et al. [27] conducted an RCT with 12 chronic post-stroke PWA, where cathodal stimulation during verb-focused therapy yielded greater improvements in verb generation than sham, though both conditions increased overall correct responses in this task and in verb oral naming. Sebastian et al. [28] conducted an RCT with 21 participants, revealing significant gains in naming untrained items immediately and two months post-treatment with real tDCS compared to sham, alongside an order-by-treatment interaction favoring tDCS-first stimulation, suggesting cerebellar engagement in language and/or skill learning. The authors [28] noted potential cathodal advantages over anodal but highlighted individual variability and the need for further studies. In a subsequent study, the same research group reported functional communication gains in an RCT of 32 participants, showing tDCS-induced enhancements in communication skills as reported by caregivers, with anodal polarity driving immediate post-treatment gains and cathodal supporting greater maintenance at two months [29]. Zheng et al. [30] found no significant improvements in primary language measures assessed with the Western Aphasia Battery in a sham-controlled trial of 47 PWA using anodal tDCS and personalized speech–language therapy, though it improved quality of life in psychosocial and physical domains, suggesting modulation of cognitive, affective, and sensorimotor processes beyond language-specific functions. However, due to methodological challenges, they suggested further studies to confirm the findings. Finally, single-case evidence in a bilingual participant [31] showed that anodal tDCS led to greater improvements in untrained picture naming and picture description compared to sham and cross-language transfer to the untreated language. Following this study, the authors reported findings in a case series involving bilingual participants with post-stroke or primary progressive aphasia [32] and concluded that cerebellar A-tDCS compared to sham led to greater enhancement of language recovery in both languages, along with gains in inhibitory control. Among the two post-stroke participants included in the case series, anodal tDCS yielded gains only in one case, underscoring the need for personalized approaches due to individual variability.

These mixed outcomes—encompassing language-related benefits like naming and spelling accuracy, functional communication (e.g., in ASHA-FACS), and broader psychosocial and physical improvements—highlight cerebellar tDCS’s potential, yet inconsistent, efficacy, often moderated by polarity, study design, task, and participant factors.

Most research focuses on Western languages and populations, with limited evidence for tonal languages like Cantonese in Chinese-speaking groups. This exploratory study reports a small-scale RCT targeting Cantonese speakers to investigate right-posterolateral cerebellar tDCS as an adjunct to aphasia rehabilitation. We employed a computerized intervention based on Fridriksson et al. [33,34,35] that has been used in previous cerebellar tDCS studies [28,29]. To expand on previous findings, this is the first study to explore grammar-specific effects of cerebellar tDCS by exposing participants to both noun- and action-naming training. Additionally, we used high-definition tDCS, shown to be as effective as conventional tDCS for enhancing treatment outcomes in chronic post-stroke aphasia, with comparable feasibility, acceptability, and efficacy outcomes when a similar computerized naming intervention was used [36]. This double-blind, randomized crossover study tests the hypothesis that A-tDCS combined with a computerized naming intervention improves naming accuracy and reduces reaction times compared to sham in Cantonese PWA. For PWA, it has been reported that anodic tDCS can enhance behavioral treatments involving nouns [25,31,37], so our preliminary study will expect to support these previous findings with more rigorous methodology (RCT). For verb naming, only cerebellar cathodal tDCS has been studied [27], so our study will explore this question for the first time. Beyond group analyses, we conducted individual analyses to better understand the effects of cerebellar tDCS in our sample.

## 2. Methods

### 2.1. Participants

Ten participants were recruited in Hong Kong through social media platforms and posters displayed at social support groups and community rehabilitation centers. Inclusion criteria were as follows: Cantonese native speakers, age between 40 and 80 years, diagnosed with aphasia, a single left-hemispheric stroke at least 6 months prior, premorbid right-handedness, normal or corrected-to-normal vision, and functional hearing acuity (with or without hearing aids). Exclusion criteria included a history of brain surgery, seizures within the past 12 months, conditions contraindicated for tDCS (e.g., metallic implants or pacemakers), prior neurological treatment (other than stroke-related), severe cognitive impairment, and naming accuracy below 10% or above 80% on a noun- and verb-naming screening pretest. The study was approved by the Human Research Ethics Committee at the University of Hong Kong prior to data collection and followed the Declaration of Helsinki. All participants and/or their caregivers provided written informed consent.

Initial screening occurred one week before the first phase of the experiment in the Speech and Neuromodulation Lab at the University of Hong Kong and consisted of completing a medical history interview, the quick version of the standardized Cantonese Aphasia Battery (Q-CAB [38]), and a computerized verb- and noun-naming test to assess suitability of the accuracy range to participate in the study. Additionally, a practice version of the computerized aphasia treatment was undertaken to familiarize the participants with the behavioral intervention.

Three participants were excluded for not meeting the accuracy criteria and one participant due to not complying with the single stroke criterium. Six eligible participants (two female, four male) with chronic post-stroke aphasia, aged 53 to 78 years (M = 66.5, SD = 8.48), were included and completed all phases of the study. The time since stroke onset ranged from 23 to 63 months (M = 46.3, SD = 14.4). Q-CAB Aphasia Quotient scores ranged from 4.04 to 6.71 (M = 5.39, SD = 0.874). All participants were diagnosed with non-fluent aphasia, with five classified as moderate and one as severe. Four participants provided MRI scans that confirmed the presence of a single ischemic cerebrovascular accident in the left hemisphere, while the medical records of the remaining two indicated similar conditions. The assigned stimulation order and the participants’ demographic and clinical information are summarized in Table 1.

### 2.2. Study Design and Outcome Measures

This study employed a randomized, double-blind, sham-controlled, crossover clinical trial design. Participants were randomly assigned to either the “tDCS-first” or “sham-first” group. Both participants and researchers administering clinical testing and treatment were blinded to the type of tDCS delivered. The data analysis was also concluded before the neuromodulation condition was revealed to the researchers. Two independent study coordinators conducted the randomization and facilitated the setup for either active or sham tDCS sessions. It is notable that the people who provided behavioral intervention and operated tDCS as well as outcome measures were blinded to the treatment conditions.

**Figure 1:**
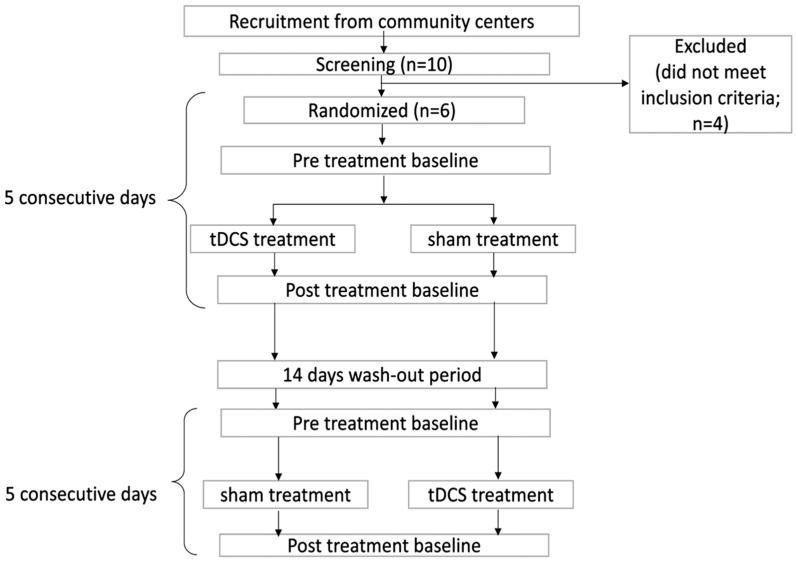
Flowchart of crossover clinical trial design.

Each treatment phase consisted of five consecutive daily sessions, in which participants received 60 min computerized naming treatment, with the first 20 min involving either active or sham tDCS. A 2-week washout period separated the two phases to minimize carryover effects (Figure 1).

The primary outcome measure was performance on a timed confrontation naming task comprising 120 picture stimuli from the Object and Action Naming Battery (OANB) [39,40] collected using the DMDX software (version 6.4.0.0) [41]. Participants were instructed to name displayed pictures as quickly and accurately as possible using a microphone, following procedures described in Momenian et al. [42]. Each trial started with a central fixation point displayed for 500 ms, followed immediately by a single picture stimulus presented centrally on the screen. The stimulus remained visible until a response was detected or 5000 ms had elapsed. DMDX recorded an error if no response was produced within the 2000 ms time limit, but all recordings were reviewed for the naming accuracy analysis.

The naming task consisted of 60 trained and 60 untrained words, matched for imageability, which is considered a strong psycholinguistic predictor for Chinese picture naming, based on norms for Cantonese speakers [42]. Naming accuracy and reaction times were recorded. These measures were collected on the first day before treatment and the final day after treatment for each phase.

### 2.3. Computerized Naming Treatment

**Figure 2:**
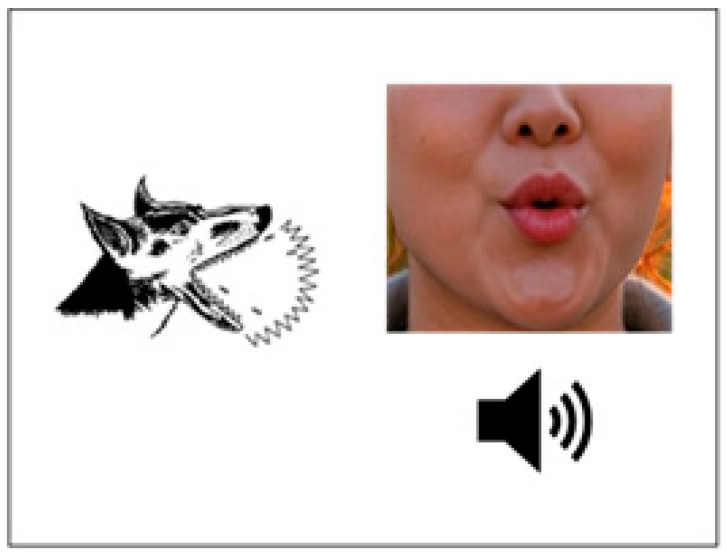
Example of the audio–visual speech perception intervention. Participants self-administered behavioral treatment, combined with active or sham transcranial direct-current stimulation. In the example, the participant is shown a picture of a wolf howling, followed by an auditory–articulatory cue (correct or incorrect action or noun name). The participant is given up to 7 s to press the “correct” or “incorrect” button in the keyboard, receiving immediate feedback.

The 60 min computerized naming treatment was adapted to Cantonese from Fridriksson et al. [33,34,35] and delivered using DMDX [41]. The treatment engaged all domains of lexical–semantic processing by providing auditory and visual articulatory cues, which has been reported to improve naming ability in post-stroke people with aphasia (e.g., [28,29,33]). The program featured 100 Cantonese picture stimuli (50 objects and 50 actions) from the OANB [39,40], 60 of which included in the outcome naming task (trained items). The trained items and untrained items (*n* = 40) have norms for timed picture naming in Cantonese [42]. Participants viewed a black-and-white image of an item for 2 s, followed by a video showing the lower part of a speaker’s face (nose and mouth) articulating the corresponding word. Participants then had up to 7 s to judge whether the picture stimulus matched the audio–visual presentation by pressing a “correct” or “incorrect” button. Each session randomly presented the trained items twice, with half of the trials featuring correct matches. The incorrect matches were randomly selected from three categories: semantic, phonological, or unrelated errors. Immediate feedback was provided after each response, displaying a “happy face” for correct answers and a “frowning face” for incorrect ones. A example of the intervention is provided in Figure 2.

### 2.4. tDCS Protocol

**Figure 3:**
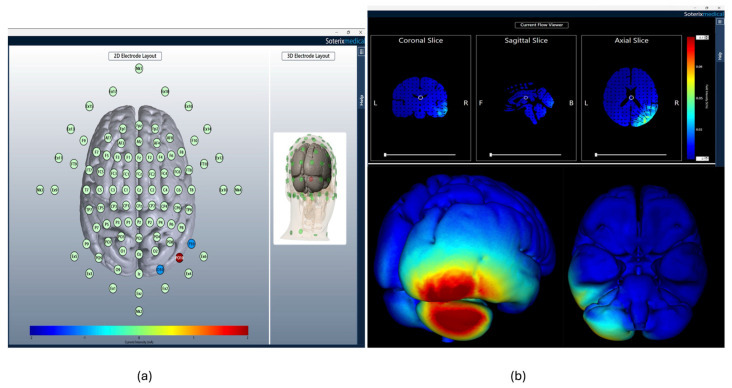
HD-tDCS montage and computer modeling of the current flow. (**a**) HD-tDCS montage configured according to the 10–20 system. Real HD-tDCS was applied to the right-posterolateral cerebellum, and one anodal electrode (2 mA) was placed at PO10, with two return electrodes (−1 mA each) positioned at P10 and O10. (**b**) Computer modeling illustrates the simulated distribution of electrical currents based on the HD-tDCS montage used in this study.

Cerebellar tDCS was delivered using a Soterix Medical MxN High-Definition tDCS Stimulator (SOTERIX MEDICAL INC, Woodbridge, NJ, USA). Real tDCS was applied to the right-posterolateral cerebellum, with one anodal electrode (2 mA) placed at PO10 and two return electrodes (−1 mA each) at P10 and O10, using EEG-sized electrodes (external diameter of 1.2 cm) secured in an EEG cap. For real tDCS, participants received 20 min of 2 mA anodal stimulation with 30 s ramp-up and ramp-down periods. For sham tDCS, electrodes were placed similarly, but the stimulator was turned off after a 30 s ramp-up and ramp-down period. Figure 3 illustrates the 10–20 system montage and computer modeling of current flow based on the HD-tDCS montage used in this study.

Participants were asked about adverse effects such as pain and/or discomfort (e.g., itching, irritation, tingling, or burning) before and after each session. The Wong–Baker FACES Pain Rating Scale [43] was used to assess any tDCS-related discomfort [44].

### 2.5. Data Analysis

Statistical analyses were conducted using R version 4.4.1 [44] to examine the effects of the intervention on naming accuracy and reaction time (RT). Responses were audio-recorded and reviewed for suitability prior to RT analysis; stimuli were excluded from the RT analysis if contaminated by extraneous sounds before naming (e.g., coughs, starters, or fillers) or when the response provided was incorrect. Additionally, RT outliers exceeding 3 standard deviations (SDs) from the participants’ means were removed. Generalized linear mixed-effects models were used to assess naming accuracy, while linear mixed-effects (LME) models were utilized to evaluate naming RT for correct responses. Both models were generated using maximum likelihood estimation. The random effects model included the random intercepts of subjects and items, and fixed effects included time (pre- vs. immediate post-treatment), grammatical category (nouns vs. verbs), treatment condition (active vs. sham), item type (trained vs. untrained), and their interactions. Pair-wise post hoc tests were conducted to further explore significant effects, with Bonferroni’s or Tukey’s corrections applied to significant results.

We employed linear mixed-effects models (LME), due to the hierarchical structure of the data and the limited sample size. This analysis is suitable for the study because our data consisted of trial-level measures (reaction time and accuracy) nested within subjects and items. Despite the small number of participants, we had a substantial number of observations (2880 for the GLMM and 1282 for the LME model, see details in the Results section). Consistent with standard practices in aphasiology, RTs were only analyzed for correct responses. Given the severity of aphasia in our sample, this resulted in the exclusion of 55.5% of the total trials. In post-stroke aphasia, heterogeneity across individuals is considerable due to lesion variability and cognitive profiles. LME addresses this by modeling random effects for subjects and items, yielding more robust estimates and reducing bias [45,46].

Carryover effects were evaluated by comparing accuracy in phase 1 post-treatment with accuracy in phase 2 pre-treatment. To examine potential order effects in the crossover design (sham-first vs. tDCS-first), naming accuracy and reaction times (RTs) were analyzed for effects of order and its interaction with time (pre- vs. post-treatment). *T*-tests were employed to compare adverse effects between the sham and active conditions.

To assess individual variability in response to treatment, we plotted performance for visual inspection of accuracy and mean RT raw scores. Additionally, McNemar’s test was used to evaluate changes in naming accuracy for each participant across time (pre- vs. immediate post-treatment), condition (active vs. sham), and grammatical category (nouns vs. verbs).

## 3. Results

### 3.1. Group Effect on Treatment Type (Real Versus Sham)

#### 3.1.1. Accuracy

**Figure 4:**
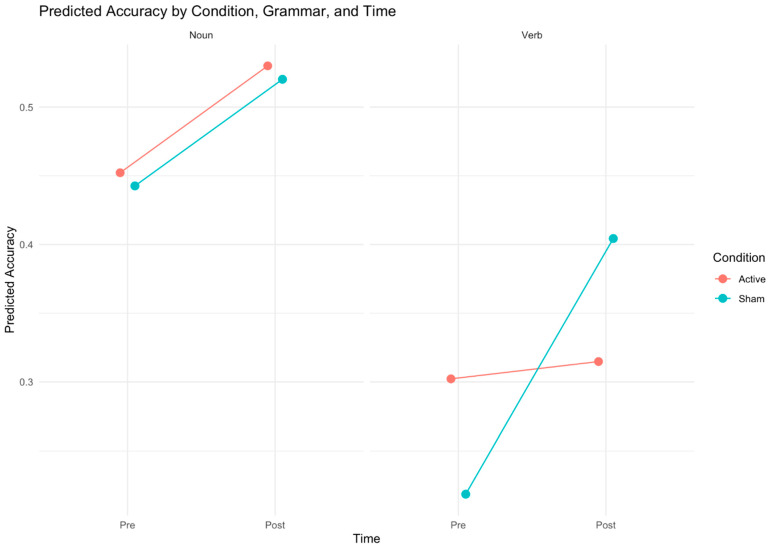
Generalized linear mixed-effects model: predicted naming accuracy by condition, grammar, and time. Note: The best-fit model revealed a significant three-way interaction between condition, grammatical category, and time (*p* = 0.035). Post hoc analyses demonstrated that following sham stimulation, verb-naming accuracy significantly improved (*p* = 0.0002). Verb–noun accuracy differences were attenuated post-sham, pointing to improvements in verb naming in this condition.

Naming accuracy on the timed confrontation naming task (120 stimuli: 60 trained, 60 untrained; 60 nouns, 60 verbs) was analyzed using a generalized linear mixed-effects model (GLMM) with random intercepts for subjects and items. The maximal model included fixed effects of time (pre- vs. post-treatment), treatment condition (active vs. sham tDCS), grammatical category (nouns vs. verbs), item type (trained vs. untrained), and their interactions. No significant main effects were found in the maximal model for time (z = 1.560, *p* = 0.119), condition (z = −0.283, *p* = 0.777), item type (z = −0.125, *p* = 0.901), or grammatical category (z = −1.679, *p* = 0.093), and most interactions were non-significant (*p* > 0.05), except for a marginal three-way interaction between condition, grammatical category, and time (z = 1.832, *p* = 0.067). The best-fit model is summarized in Table 2. It excluded the non-significant variables and their interactions (i.e., item type and its interaction with other variables) and revealed a significant main effect of grammatical category (z = −2.604, *p* = 0.009), with nouns showing higher accuracy than verbs, and a significant three-way interaction between condition, grammatical category, and time (z = 2.114, *p* = 0.035) (see Figure 4). Pairwise comparisons with Bonferroni’s correction indicated that post-treatment verb-naming accuracy significantly outperformed pre-treatment verb-naming accuracy in the sham condition (z = −0.200, *p* = 0.0002). However, in active tDCS, no significant improvement in naming accuracy was found for nouns (z = −1.600, *p* > 0.99) and verbs (z = −0.301, *p* > 0.99). In the sham condition, pre-treatment naming accuracy in nouns was significantly higher than in verbs (z = 4.175, *p* = 0.0008), but no significant differences were found post-treatment between nouns and verbs (z = 1.893, *p* > 0.99). On the other hand, in the active condition, no significant difference was found between noun- and verb-naming accuracy pre-treatment scores (z = 2.604, *p* = 0.2583), but post-treatment accuracy of nouns was significantly higher than verbs (z = 3.612, *p* = 0.0085). Other pairwise contrasts were non-significant (*p* > 0.05).

#### 3.1.2. Reaction Times

Of the 2880 naming accuracy data points (120 stimuli: 60 trained, 60 untrained; 60 nouns, 60 verbs; 6 subjects), 1282 reaction time (RT) data points for correct responses were analyzed using a linear mixed-effects model (LME), with random intercepts for subjects and items, after excluding 55.5% of trials due to incorrect responses and outliers responses exceeding 3 SD from each participant’s mean; a summary is presented on Table 3. Despite the exclusion rate, the analysis retained 644 observations for the active condition, 638 observations for the sham condition, and a sufficient number of observations across grammatical categories, specifically 720 for nouns (with 362 in active tDCS) and 562 for verbs (with 358 in active tDCS), ensuring convergence in LME models. The maximal model included fixed effects of time (pre- vs. post-treatment), condition (active vs. sham tDCS), grammatical category (nouns vs. verbs), item type (trained vs. untrained), and their interactions. Only the interaction between grammatical category and item type was significant (t = 2.028, *p* = 0.043), indicating longer RTs for untrained verbs compared to untrained nouns (estimate = 533.155 ms, SE = 262.856). No significant main effects were found for time (t = 0.913, *p* = 0.361), condition (t = −0.260, *p* = 0.795), grammatical category (t = 1.490, *p* = 0.137), or item type (t = −0.048, *p* = 0.962), and other interactions were non-significant (*p* > 0.05). The best-fit model, summarized in Table 3, retained grammatical category and time and revealed a significant main effect of grammatical category (t = 4.008, *p* < 0.001), with verbs eliciting longer RTs than nouns (estimate = 402.597 ms, SE = 100.458), and there was a significant interaction between grammatical category and time (t = −2.303, *p* = 0.021), indicating greater RT reduction for verbs post-treatment compared to nouns (estimate = −284.289 ms, SE = 123.433). Post hoc comparisons showed that pre-treatment noun RTs were significantly faster than verb RTs (estimate = −403 ms, SE = 101.0, *p* = 0.0005), while no significant difference was observed post-treatment (estimate = −118 ms, SE = 94.3, *p* = 0.593). Within-category comparisons showed no significant RT changes for nouns (pre- vs. post-treatment: estimate = −113 ms, SE = 81.5, *p* = 0.511) or verbs (pre- vs. post-treatment: estimate = 172 ms, SE = 92.8, *p* = 0.250). These findings suggest that the computerized naming intervention attenuated pre-treatment RT differences between nouns and verbs by reducing verb RTs more substantially. The lack of significant condition or item type effects in the final model suggests that tDCS and training status had minimal impact on RTs.

#### 3.1.3. Order Effects

LME and GLMM analyses were conducted to examine the order effects on naming accuracy and RT. The fixed variables included in the model were treatment order (sham–active vs. active–sham) and time (pre vs. post). For naming accuracy, no significant main effects of order (z = −1.404, *p* = 0.160) or order-by-time interaction (z = −0.562, *p* = 0.574) were found. Similarly, no significant main effects of order (t = 0.323, *p* = 0.760) or order-by-time interaction (t = −1.491, *p* = 0.136) were found for RT. These findings suggest that whether participants received sham or tDCS first did not significantly affect naming accuracy or RTs, supporting the reliability of the crossover design.

### 3.2. Individual Variability

**Figure 5:**
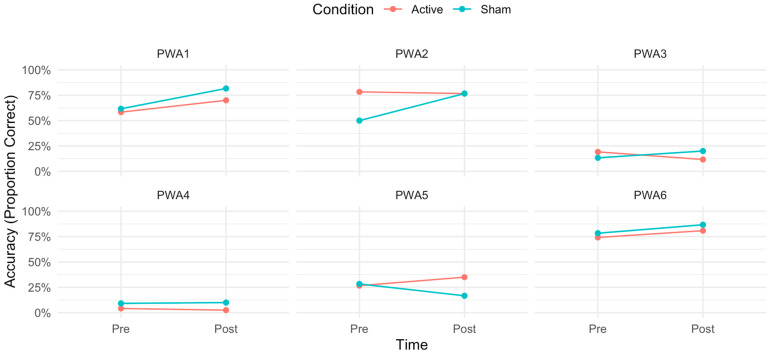
Individual variability in overall naming accuracy by condition. Note: Responses to treatment were highly heterogeneous. Anodal tDCS conferred clear benefits in only one participant (PWA5). Notably, PWA2 and PWA3 showed larger verb-naming gains under sham than anodal stimulation, a pattern that suggests possible inhibition of verb retrieval by A-tDCS in this subgroup. PWA1 and PWA6 improved significantly in both conditions (no tDCS effect), whereas PWA4 showed no significant change overall, except in the stratified analysis by word class (nouns vs. verbs), which revealed significant gains in verb naming in S-tDCS, see Figure 6.

Individual variability in naming accuracy was assessed using McNemar’s test to evaluate changes in correct responses pre- to post-treatment across conditions (A-tDCS vs. S-tDCS) and word classes (nouns vs. verbs) (Figure 5). In the A-tDCS condition, significant improvements in overall accuracy were observed for PWA1 (χ^2^ = 24.0, *p* < 0.001), PWA5 (χ^2^ = 10.00, *p* = 0.002), and PWA6 (χ^2^ = 8.00, *p* = 0.005) but not for PWA2 (χ^2^ = 2.00, *p* = 0.157) or PWA4 (χ^2^ = 2.00, *p* = 0.157). PWA3 showed a significant decrease in overall accuracy (χ^2^ = 9.00, *p* = 0.003). In the S-tDCS condition, significant improvements were seen for PWA1 (χ^2^ = 12.3, *p* < 0.001), PWA2 (χ^2^ = 32.0, *p* < 0.001), PWA3 (χ^2^ = 8.00, *p* = 0.005), and PWA6 (χ^2^ = 10.00, *p* = 0.002) but not for PWA4 (χ^2^ = 1.00, *p* = 0.317). PWA5 showed a significant decrease in overall accuracy (χ^2^ = 14.0, *p* < 0.001).

**Figure 6:**
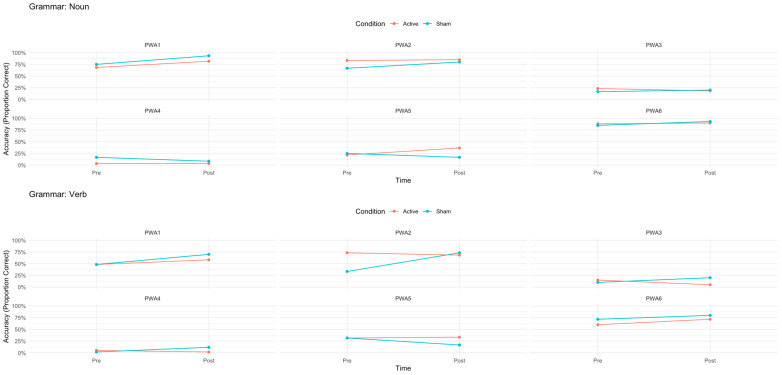
Individual variability in noun and verb naming accuracy by condition. Note: A dissociation between nouns and verbs post-treatment is noted. PWA5 showed significantly improved naming accuracy only in anodal tDCS, driven by significant gains in noun naming. In contrast, PWA2 and PWA3 improved after S-tDCS but not A-tDCS, driven by gains in mainly in verb naming that occurred in sham. For PWA3, this contrasts with significant decline in verbs in A-tDCS. PWA4 did not show changes in overall accuracy; however, when verbs were analyzed separately, significant improvement in verbs was observed in the sham condition only.

For nouns and verbs separately, patterns were also varied (Figure 6). PWA1 showed significant improvements in both conditions for nouns (A-tDCS: χ^2^ = 8.00, *p* = 0.005; S-tDCS: χ^2^ = 11.0, *p* < 0.001) and verbs (A-tDCS: χ^2^ = 4.50, *p* = 0.034; S-tDCS: χ^2^ = 11.3, *p* < 0.001). PWA2 showed no significant changes in A-tDCS (nouns: χ^2^ = 2.00, *p* = 0.337; verbs: χ^2^ = 4.00, *p* = 0.280) but significant improvements in S-tDCS for nouns and verbs (nouns: χ^2^ = 8.00, *p* = 0.005; verbs: χ^2^ = 24.0, *p* < 0.001). PWA3 showed non-significant changes for nouns in both conditions (A-tDCS: χ^2^ = 6.00, *p* = 0.265; S-tDCS: χ^2^ = 3.00, *p* = 0.062) but a significant decline in verbs in A-tDCS (χ^2^ = 6.00, *p* = 0.014) and significant improvements in verbs in S-tDCS (χ^2^ = 6.00, *p* = 0.014). PWA4 showed no significant changes in A-tDCS (nouns: χ^2^ = 1.00, *p* = 0.398; verbs: χ^2^ = 3.00, *p* = 0.093) but a significant decline in S-tDCS for nouns (χ^2^ = 5.00, *p* = 0.025) and a significant improvement for verbs (χ^2^ = 6.00, *p* = 0.014). PWA5 showed significant improvements for nouns in A-tDCS (χ^2^ = 9.00, *p* = 0.003) but not for verbs (χ^2^ = 2.00, *p* = 0.178), and in S-tDCS, there were significant benefits for verbs (χ^2^ = 9.00, *p* = 0.003) and a significant decline for nouns (χ^2^ = 5.00, *p* = 0.025). PWA6 showed non-significant changes for nouns in A-tDCS (χ^2^ = 2.00, *p* = 0.207) but significant improvements for verbs (χ^2^ = 7.00, *p* = 0.008) and for both in S-tDCS (nouns: χ^2^ = 5.00, *p* = 0.025; verbs: χ^2^ = 5.00, *p* = 0.025).

### 3.3. Adverse Effects

Most of the participants reported a slight increase in discomfort level for active conditions, with only one participant (PWA6) rating higher discomfort in S-tDCS. There was no significant difference in pain level (*p* = 0.87) between real (*M* = 3.13, *SD* = 2.58, *SE* = 0.47) and sham conditions (M = 2.93, *SD* = 1.72, *SE* = 0.31). This finding indicates that the individuals found the treatment tolerable and that the blinding effect was effectively maintained.

### 3.4. Carryover Effects

No significant differences in naming accuracy were observed in the comparison of the post-treatment phase 1 and pre-treatment phase 2 accuracy (df = 4, *p* = 0.33) between real–sham (M = 6.17, SE = 2.32, SD = 4.10) and sham–real (M = 3.2, SE = 1.35, SD = 2.34) groups, indicating no carryover effect.

## 4. Discussion

This study evaluated the efficacy of five sessions of 2 mA anodal transcranial direct-current stimulation (A-tDCS) targeting the right-posterolateral cerebellum as an adjunct to a computerized naming intervention for improving noun and verb oral naming in Cantonese-speaking individuals with chronic post-stroke aphasia. Using a double-blind, randomized crossover design, we devised a Chinese version of an audio–visual speech perception intervention [33,34,35], previously used in cerebellar tDCS studies for post-stroke aphasia [28,29]. Unlike previous cerebellar tDCS studies that focused exclusively on nouns or verbs, the naming training protocol and outcome measures incorporated both nouns and verbs. To our knowledge, this is the first study to directly compare the effects of cerebellar tDCS on timed picture-naming performance for these two grammatical classes.

Contrary to our hypothesis that right-posterolateral cerebellar A-tDCS would contribute to higher performances on naming accuracy and to faster reaction times (RTs) compared to sham tDCS (S-tDCS), group-level analysis revealed that this protocol did not enhance naming performance beyond the behavioral intervention alone. In fact, S-tDCS produced a significantly greater improvement in verb naming accuracy than A-tDCS, revealed by a significant interaction between condition, time, and grammatical class (see Figure 4). Additionally, a separate significant interaction between grammatical category and time suggests that the computerized naming intervention reduced pre-treatment RT differences between nouns and verbs, primarily by decreasing verb RTs, independent of tDCS. To further understand the source of the interaction and acknowledging the exploratory nature of the study, individual participant analyses were conducted, and they revealed substantial inter-individual variability (Figure 5 and Figure 6). PWA5 was the only participant who showed clear benefit from A-tDCS, with significant accuracy gains confined to the active condition and driven by nouns. In contrast, PWA2 and PWA3 improved significantly only in S-tDCS, with larger verb-naming gains. Notably, PWA3 also showed a significant decline in verb accuracy under A-tDCS. PWA4 exhibited no overall accuracy change but, when analyzed separately by grammatical class, demonstrated significant verb improvement exclusively in S-tDCS. Finally, PWA1 and PWA6 improved significantly in both conditions, indicating benefit from the behavioral intervention alone or practice effects with no differential tDCS effect.

Taken together, these findings suggest that anodal tDCS over the right-posterolateral cerebellum may have exerted an inhibitory effect on the mechanisms of learning triggered by audio–visual speech perception training (at least in some cases), specifically for verbs. Whereas noun retrieval appeared relatively unaffected or slightly facilitated in isolated cases, verb retrieval showed greater gains under sham stimulation in three of the six participants, with an actual decline under A-tDCS in PWA3. The findings highlight the critical influence of stimulation parameters such as polarity, along with individual functional factors on outcomes and underscores the need for larger trials that systematically investigate them.

When comparing our findings with prior cerebellar tDCS studies in post-stroke aphasia, the absence of a group-level benefit from A-tDCS is consistent with previous reports [26,30], whereas the clear benefit observed in one participant (PWA5) aligns with positive single-case or small-series outcomes [25,31,32]. Some methodological differences may contribute to these mixed results. First, the studies reporting robust A-tDCS effects employed 9–15 sessions [25,31,32], whereas our protocol was limited to 5 sessions. Although repeated stimulation is considered important for inducing lasting modulation of cerebrocerebellar circuits (e.g., via long-term depression of Purkinje cells), [28] some positive effects have been observed with five sessions [25,28]. In the present study, the significant time × grammatical class interactions for both accuracy and reaction times, together with significant individual-level improvements, confirm that behavioral gains were observable after five sessions of intensive naming therapy. Second, the nature of the behavioral intervention differed across studies. Unlike protocols that required overt speech production or written output [25,31,32], our computerized training relied on observation of audio–visual articulatory movements without enforced spoken output, which has already proven to be effective for people with aphasia (PWA) [33,35,36]. We selected this approach because of its methodological rigor, which provided greater control and less variation between sham and active tDCS conditions. Despite the absence of overt production, the intervention itself proved effective, especially for verb naming under sham stimulation.

Our results showed no significant effect of training status (trained vs. untrained items) or interactions involving training status, indicating equivalent generalization across conditions. This contrasts with a previous study [28] that reported greater improvements on untrained items with active (A-tDCS or C-tDCS) compared to sham stimulation, which the authors attributed to enhanced generalization of compensatory strategies via long-term depression (LTD). The absence of order effects in our crossover design further differs from that study [28], in which an order-by-treatment interaction for C-tDCS was reported. Other factors such as polarity or individual variability in residual cerebrocerebellar connectivity are likely to have played a role and should be systematically investigated in future trials. Language-specific neuroplasticity may also contribute to these discrepancies. The distinct morphology, grammatical markers, and orthography of Chinese (especially Cantonese) engage different neural correlates of language processing compared to alphabetic languages [47,48,49,50]. Although a direct link between these linguistic features and tDCS responsiveness remains to be established, they may partly explain the variable outcomes across studies conducted in different languages.

The greater verb-naming gains observed in the sham condition compared to A-tDCS raise the possibility of polarity-specific effects on cerebellar involvement in language recovery. This pattern is consistent with previous reports showing superior verb-related outcomes with cathodal rather than anodal stimulation over the cerebellum. Specifically, Marangolo et al. [27] found that cathodal tDCS enhanced verb generation, and Sebastian et al. [28] reported polarity-specific effects, with better overall naming accuracy under cathodal stimulation (compared with anodal or sham) in within-group analyses. C-tDCS may facilitate language performance by inhibiting Purkinje cells, thereby reducing cerebellar outflow inhibition and disinhibiting connected frontal regions [27,28,51]. However, unlike the typical pattern where anodal tDCS enhances cortical excitability and cathodal tDCS decreases it, some studies suggest that cerebellar anodal and cathodal tDCS do not reliably follow this dichotomy due to the complex folding of the cerebellar cortex [52,53]. Because we tested only anodal tDCS, we cannot determine optimal polarity, but the superior verb gains under sham in several participants suggest that our anodal protocol may have interfered with training-induced improvements in action-related naming, possibly via excessive activation of cerebellar nodes supporting verb and action-semantics processing. Another possibility is that A-tDCS created a homeostatic blocking of learning for verbs, since the cerebellum is particularly involved in sequencing and temporal dynamics, which are especially relevant for verbs/actions. The hypotheses mentioned above require confirmation in larger trials that directly contrast cerebellar anodal and cathodal stimulation, grammar-specific effects, and the impact of cerebellar neuromodulation on fine temporal processing relevant to language production.

### 4.1. Individual Variability

Individual variability significantly influenced our study’s outcomes, particularly given our small sample size, consistent with prior tDCS studies in post-stroke aphasia [28,32] and the broader tDCS literature [47,48,49]. Anatomical differences, such as lesion site, skull thickness, and scalp–cortex distance, may alter electric field distribution during cerebellar tDCS, leading to inconsistent stimulation effects [50]. Participant characteristics, including age, education, and aphasia severity, influence recovery rates [54]; notably, the participant with the lowest overall response, PWA4, presented the most severe aphasia, consistent with observations that residual language capacity predicts aphasia recovery and tDCS response [28,54]. A limitation of this exploratory study is that we did not conduct functional neuroimaging exams (such as fMRI or EEG frequency analysis) before and after the intervention to further explore the neuroanatomical correlates of the intervention effects. Future research should investigate optimized stimulation protocols, including polarity and anatomical targeting, to enhance clinical efficacy.

### 4.2. Behavioral Intervention Effects

Although not designed to test behavioral intervention’s efficacy, our study found notable grammar-specific post-treatment effects, attenuating noun–verb differences in accuracy and RT. Significant gains in action-naming accuracy occurred in the S-tDCS condition, and verb RTs decreased independent of tDCS condition. Verbs, which are more complex than nouns and more severely impaired in non-fluent aphasia [55], benefited more from the computerized visual speech perception naming intervention [33,34,35] for Cantonese speakers. This intervention engages lexical–semantic processing and activates the motor speech system without requiring expressive output, allowing multiple items to be trained in a single session without excessive patient fatigue [33]. Our results suggest that this behavioral approach is a promising alternative for improving verb naming in non-fluent aphasia, though further investigation is needed as the present study did not include a separate control group (e.g., a no-intervention aphasia group), which limits the ability to differentiate post-treatment effects from potential practice or learning effects across sessions.

### 4.3. Limitations and Future Directions

This study has several limitations. The small sample size limits statistical power and generalizability, highlighting the need for larger, more homogeneous cohorts in future research. Additionally, a high exclusion rate of reaction time data (55.5% due to incorrect responses and outliers) substantially reduced the number of observations available for RT analyses, which may have obscured subtle tDCS-related effects.

Nevertheless, the cerebellar tDCS literature in aphasia remains sparse and relies predominantly on case reports or very small series [25,26,27,28,29,30,31,32]. Therefore, despite its exploratory nature, the present double-blind, sham-controlled, randomized crossover RCT using a rigorous methodology, a within-subject design, and appropriate mixed-effects modeling (GLMM/LMM) represents a meaningful contribution to this emerging field. The grammar-specific effects evidenced point to new questions to be addressed in this area.

Other areas of improvement to the current protocol are that we assessed outcomes immediately after each treatment block with only one post-treatment session, which may have been influenced by fatigue and limits conclusions about effect stability. Additionally, the short five-session protocol and exclusive use of anodal polarity also restrict broader conclusions, although significant behavioral gains were observed in individual cases and in prior studies using similar protocols [25,28]. Given the mixed evidence on cerebellar tDCS polarity in both aphasia [28] and healthy participants [24], future studies should directly contrast anodal and cathodal stimulation.

Future research should evaluate longer-term retention of naming gains, their impact on functional communication and quality of life [28,29], and potential concurrent changes in executive functions. Larger trials incorporating neuroimaging or electrophysiological measures and detailed profiling of individual factors (lesion location, aphasia severity, baseline performance) are needed to identify predictors of response and optimize personalized cerebellar tDCS protocols for post-stroke aphasia.

## 5. Conclusions

This study provides a preliminary understanding of anodal cerebellar tDCS on noun and verb recovery in PWA. Our findings suggest that five sessions of A-tDCS targeting the right-posterolateral cerebellum did not consistently alter naming performance in Cantonese-speaking PWA, with verb improvements being more pronounced in the S-tDCS condition and with high individual variability. The lack of verb-naming improvement (or reduced gains) with A-tDCS compared to S-tDCS in Cantonese-speaking PWA may point to grammar-specific polarity effects of cerebellar stimulation, although we refrain from drawing firm conclusions from null results. This compelling issue merits further investigation in future studies using different polarity-specific protocols. Larger, multicenter trials incorporating both anodal and cathodal polarities, diverse languages, and neuroimaging are needed to optimize protocols and clarify the role of cerebellar tDCS in aphasia recovery. Such efforts could enhance therapeutic options for PWA, improving functional outcomes and quality of life. Lastly, this study demonstrated promising effects of our developed computerized audio–visual perceptual training for Cantonese-speaking people with aphasia, which can be used in future studies.

## Figures and Tables

**Table 1:** Assigned stimulation order and participants’ demographic and clinical characteristics.

ID	Stimulation Order	Age	Sex	Post-Stroke Time (Months)	Lesion Location	Q-CAB (Aphasia Severity)	Naming Screening Accuracy (%)
PWA1	tDCS first	78	F	42	Left putamen	5.71 (moderate)	58.33
PWA2	Sham first	64	M	40	External capsule and temporoparietal lobe	5.51 (moderate)	60.00
PWA3	Sham first	69	M	23	Left putamen	5.40 (moderate)	10.84
PWA4	Sham first	65	M	63	Left putamen	4.04 (severe)	15.00
PWA5	tDCS first	53	F	53	Non-specified left-hemispheric	5.01 (moderate)	33.30
PWA6	tDCS first	69	M	57	Non-specified left-hemispheric	6.71 (moderate)	73.00

Note: PWA = person with aphasia; F = female; M = male; Q-CAB = Cantonese Quick Aphasia Battery (score range 0–10, with severity classification in parentheses); pre-treatment screening accuracy reflects baseline performance on naming screening test, one week before the treatment.

**Table 2:** Summary of the generalized linear mixed-effects model for naming accuracy.

**Fixed Effects**	**Estimate**	**Std_Error**	**z_Value**	***p*** **_Value**	**CI_Lower**	**CI_Upper**
(Intercept)	−0.192	0.719	−0.267	0.790	−1.602	1.218
Condition: Sham vs. Active	−0.039	0.194	−0.200	0.842	−0.419	0.342
Grammar: Verb vs. Noun	−0.645	0.248	−2.604	0.009	−1.130	−0.159
Time: Post vs. Pre	0.312	0.195	1.600	0.110	−0.070	0.694
Condition Sham:Grammar Verb	−0.398	0.277	−1.436	0.151	−0.940	0.145
Condition Sham:Time Post	0.000	0.275	−0.002	0.999	−0.540	0.539
Grammar Verb:Time Post	−0.253	0.276	−0.917	0.359	−0.794	0.288
Condition Sham:Grammar Verb:Time Post	0.827	0.391	2.114	0.035	0.060	1.594
**Random Effects**	**Variance**	**Std_Dev**				
Item (Intercept)	0.8	0.9				
Subject (Intercept)	1.7	1.3				

**Table 3:** Summary of linear mixed-effects model for reaction times.

**Fixed Effects**	**Estimate**	**Std_Error**	**t_Value**	**DF**	***p*** **_Value**	**CI_Lower**	**CI_Upper**
(Intercept)	1783.379	102.220	17.447	9.813	0.000	1583.032	1983.726
Grammar (Verb vs. Noun)	402.597	100.458	4.008	278.948	0.000	205.703	599.491
Time (Post vs. Pre)	112.646	81.510	1.382	1186.256	0.167	−47.110	272.402
Grammar: Verb: Time Post	−284.289	123.433	−2.303	1192.987	0.021	−526.213	−42.365
**Random Effects**	**Variance**	**Std_Dev**					
Item (Intercept)	52,433	229					
Subject (Intercept)	32,476	180					
Residual	1,182,568	1087					

## Data Availability

The original contributions presented in the study are included in the article, further inquiries can be directed to the corresponding author.

## Abbreviations

The following abbreviations are used in this manuscript:

tDCS	Transcranial Direct-Current Stimulation
A-tDCS	Anodal Transcranial Direct-Current Stimulation
C-tDCS	Cathodal Transcranial Direct-Current Stimulation
S-tDCS	Sham Transcranial Direct-Current Stimulation
HD-tDCS	High-Definition Transcranial Direct-Current Stimulation
PWA	Persons/People with Aphasia
ASHA-FACS	American Speech–Language–Hearing Association Functional Assessment of Communication Skills
OANB	Object and Action Naming Battery
Q-CAB	Quick Cantonese Aphasia Battery
CSF	Cerebrospinal Fluid
MRI	Magnetic Resonance Imaging
RT	Reaction times
GLMM	Generalized Linear Mixed-Effects Model
LME	Linear Mixed-Effects Model
